# Montelukast induces beneficial behavioral outcomes and reduces inflammation in male and female rats

**DOI:** 10.3389/fimmu.2022.981440

**Published:** 2022-09-06

**Authors:** Ira S. Rostevanov, Batya Betesh-Abay, Ahmad Nassar, Elina Rubin, Sarit Uzzan, Jacob Kaplanski, Linoy Biton, Abed N. Azab

**Affiliations:** ^1^ Department of Clinical Biochemistry and Pharmacology, Faculty of Health Sciences, Ben-Gurion University of the Negev, Beer-Sheva, Israel; ^2^ Department of Nursing, School for Community Health Professions, Faculty of Health Sciences, Ben-Gurion University of the Negev, Beer-Sheva, Israel

**Keywords:** behavior, depression, inflammation, leukotrienes, mania, mental disorders, montelukast, leukotrienes-modifying agents

## Abstract

**Background:**

Accumulative data links inflammation and immune dysregulation to the pathophysiology of mental disorders; little is known regarding leukotrienes’ (LTs) involvement in this process. Circumstantial evidence suggests that treatment with leukotriene modifying agents (LTMAs) such as montelukast (MTK) may induce adverse neuropsychiatric events. Further methodic evaluation is warranted.

**Objective:**

This study aims to examine behavioral effects, as well as inflammatory mediator levels of chronic MTK treatment in male and female rats.

**Methods:**

Depression-like phenotypes were induced by exposing male and female rats to a chronic unpredictable mild stress (CUMS) protocol for four weeks. Thereafter, rats were treated (intraperitoneally) once daily, for two weeks, with either vehicle (dimethyl sulfoxide 0.2 ml/rat) or 20 mg/kg MTK. Following treatment protocols, behavioral tests were conducted and brain regions were evaluated for inflammatory mediators including tumor necrosis factor (TNF)-α, interleukin (IL)-6 and prostaglandin (PG) E2.

**Results:**

Overall, MTK did not invoke negative behavioral phenotypes (except for an aggression-inducing effect in males). Numerous positive behavioral outcomes were observed, including reduction in aggressive behavior in females and reduced manic/hyperactive-like behavior and increased sucrose consumption (suggestive of antidepressant-like effect) in males. Furthermore, in control males, MTK increased IL-6 levels in the hypothalamus and TNF-α in the frontal cortex, while in control females it generated a robust anti-inflammatory effect. In females that were subjected to CUMS, MTK caused a prominent reduction in TNF-α and IL-6 in brain regions, whereas in CUMS-subjected males its effects were inconsistent.

**Conclusion:**

Contrary to prior postulations, MTK may be associated with select beneficial behavioral outcomes. Additionally, MTK differentially affects male vs. female rats in respect to brain inflammatory mediators, plausibly explaining the dissimilar behavioral phenotypes of sexes under MTK treatment.

## Introduction

Mental illness causes staggering burden to millions worldwide ensuing tremendous healthcare expenditures and hardship to patients and their families (1). Associated with distress and impairment of personal functioning, psychiatric disorders impact mood, cognition, behavior, and can lead to suicidal attempts and death (2, 3). Though plausibly multifactorial, the exact etiology of mental disorders and the underlying pathophysiological mechanisms are poorly understood.

More and more studies have examined immune disturbance, inflammatory processes and mental illnesses, particularly mood disorders (4–7). Though the direct intermediating immune-pathogenic mechanisms are still unofficially established, interactions between the immune system and the brain have attracted considerable attention in the field of neuropsychiatric diseases (8–10), and brain regions including the frontal cortex (FC), hippocampus (HC) and hypothalamus (HT) have been repetitively linked to such (11–13). Inflammatory mediators (such as prostaglandin [PG] E2, interleukin [IL]-6, and tumor necrosis factor [TNF]- α), which regulate brain function, proliferation, differentiation, and survival of brain cells, have also shown interconnection to psychiatric disorders (5, 14). To this end, pharmacotherapeutic strategies targeting neuroinflammatory components have been arduously explored in quest to further effective medicinal approaches to mental illness (15–26); this pursuit is of particular pertinence given the untoward side-effects, low adherence rates and limited positive outcomes associated with most available psychiatric medications today (2, 27).

**Figure 1:**
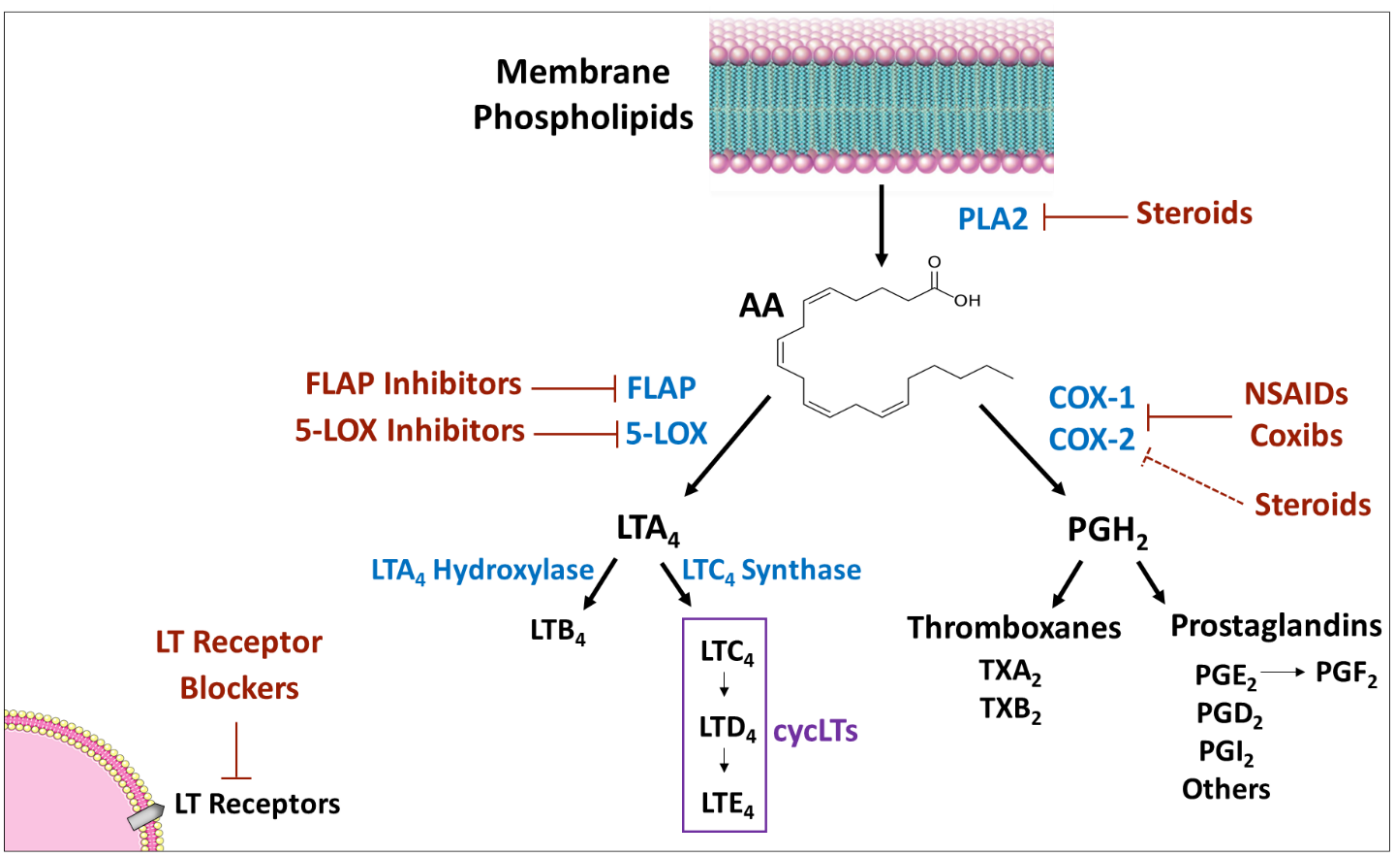
Eicosanoids synthesis cascade. PLA2 produces AA from membrane phospholipids. Thereafter, there are two main pathways for AA metabolism: 1) COX pathway - the enzymes COX-1 and 2 convert AA to PGH2 which is converted by different isozymes to various prostaglandins and thromboxanes; and, 2) 5-LOX pathway - the enzyme 5-LOX (together with the enzyme FLAP) converts AA to LTA4, which is then converted to various leukotrienes. Medications that influence the synthesis/activity of mediators/enzymes are marked in red. Arrow indicates “production/induction”; solid line indicates inhibition/locking; dashed line indicates indirect inhibition. AA, arachidonic acid; COX, cyclooxygenase; FLAP, five lipoxygenase activating protein; LOX, lipoxygenase; LT, leukotriene; NSAIDs, non-steroidal anti-inflammatory drugs; PG, prostaglandin; PL, phospholipase; TX, thromboxane.

Leukotrienes (LTs) are inflammatory mediators eventuating from the phospholipids-arachidonic acid (AA)-eicosanoids pathway in mammalian tissues. In this pathway, AA – a polyunsaturated fatty acid – is metabolized to eicosanoids, including prostanoids (PGs and thromboxanes) and LTs (see **Figure 1** for illustration). AA is converted by the enzyme cyclooxygenase to PGH2 – the precursor from which all prostanoids are produced. Similarly, AA is transformed by the enzyme 5-lipoxygenase (5LOX) [and the enzyme 5LOX-activating protein (FLAP)] to LTA4 – the precursor from which all other LTs are produced. Several anti-inflammatory and allergy medications work by altering various junctions in the phospholipids-AA-eicosanoids cascade, such as nonsteroidal anti-inflammatory drugs (NSAIDs), corticosteroids, inhibitors of 5LOX and FLAP (**Figure 1**).

LTs strongly affect function of immune cells and play important roles in allergic, cancerous, respiratory, cardiovascular and inflammatory diseases (28, 29). Leukotrienes-modifying agents (LTMAs) are regarded as one of the most important drug families used clinically for the treatment of inflammation-and-allergy-related disorders (30). Among this drug class, montelukast (MTK) is a prime medication commonly prescribed for maintenance treatment of asthma, exercise induced bronchospasm, allergic rhinitis, and urticaria (28, 31, 32). MTK works mainly by blocking cysteinyl leukotriene (cys-LT) receptors in the lungs resulting in decreased inflammation and relaxation of smooth muscles (33–35). However, MTK purportedly exerts additional mechanisms of action. It alters several cellular signaling pathways [such as the cyclic adenosine monophosphate (cAMP)-extracellular signal-regulated kinase (ERK)] and possesses various immune-modulating and anti-inflammatory characteristics, including suppression of leukocyte proliferation and migration, attenuation of pro-inflammatory mediators (e.g., IL-6, IL-8, TNF-α and PGE2), inhibition of nuclear factor-κ B function, and more (36–44). As abovementioned, research shows an iterated correlation between inflammatory biomarkers and psychiatric disorders. For example, several studies demonstrated a distorted Th1/Th2 immune response in patients with major depression (45, 46). Thus, given the aforementioned secondary immune-modulating and anti-inflammatory mechanisms of MTK, it is logical that MTK would impact behavioral outcomes. Nonetheless, it is important to bear in mind that the alteration of the Th1/Th2 adaptive immune response by MTK (47, 48) may conceivably give way to adverse outcomes. Disbalance of the Th1/Th2 immune response may impair the defensive mechanisms against specific viral, bacterial and parasitic pathogens, thus, potentially increasing the incidence of opportunistic infections.

Reports show LT inhibitors ascertaining only modest therapeutic efficacy (49, 50). Suggested explanations for this include: individual differences in LT levels, heterogeneity of disease phenotypes, and, differences in drug pharmacokinetics, pharmacodynamics and pharmacogenomics (51–55). Another possible rationalization is sex-related differences in medication response, possibly relating to the impact of androgen levels on LTMA mediation (56), as well as the female sex hormones relating to allergic manifestations (57). In this context, a major limitation of many preclinical pharmacological studies is the exclusive inclusion of only male animals, neglecting half of the possible consumers of pharmacological treatments (51–54, 58–63). This underscores the necessity to evaluate effects of LTMAs (such as MTK) in both male and female subjects.

Almost nothing is known about the involvement of LTs in the pathogenesis of mental illness. Post-marketing reports and pharmacovigilance studies suggested that the use of LTMAs, and MTK, in particular, may be associated with the development of various adverse neuropsychiatric events (ANPEs), such as depression, aggression, suicidal ideation, anxiousness, hallucinations, sleep disturbances, irritability, tremors, and restlessness (32, 64–74). For example, a recent large retrospective, propensity score-matched cohort study by Paljarvi *et al.* (73) demonstrated that MTK treatment was associated with increased incidence of ANPEs among patients with asthma and allergic rhinitis. There was a notably high odds ratio for anxiety disorders among asthmatic patients treated with MTK, and a high odds ratio for insomnia in MTK-treated patients with allergic rhinitis (73). However, most of the evidence regarding ANPEs and MTK is circumstantial and not causative, based mainly on retrospective study models (65, 70, 71). Stratified analyses suggest that the reported increased incidence in ANPEs may instead relate to the underlying diseases for which MTK is being administered (e.g., asthma and allergic rhinitis), rather than MTK treatment itself (65, 71).

Though anecdotal and unestablished reports show that LTMAs (MTK in particular) may induce ANPEs, we hypothesized that given its immune-modulating and anti-inflammatory effects, MTK might potentiate *beneficial* behavioral effects in patients with psychiatric diseases (31, 32, 75–78). Taking into account the reports of adverse behavioral effects of MTK (70, 71, 79), we recognized that using MTK as treatment for psychiatric disorders may sound problematic. However, given the essence of the adverse data, and considering anteceding paradigms in medicine where an absolute contraindicated treatment transitioned to become mainstream (for example, β-adrenergic receptor antagonists for chronic heart failure) (80), we discretionarily concluded it was an advantageous undertaking.

This study aims to examine the behavioral effects of chronic MTK treatment in male and female rats using different animal behavioral models, as well as determine the effects of chronic MTK treatment on brain inflammatory mediator levels using pharmacological studies in male and female rats.

## Materials and methods

### Animals

Male and female Sprague-Dawley rats approximately eight weeks of age, weighing 220-250 gr (males) or 180-200 gr (females) at the beginning of the experiments were used throughout the studies. Housing comprised of three rats per cage and environmentally-regulated conditions (ambient temperature 22 ± 1°C, relative humidity 55–58%, and photoperiod cycle 12 h light: 12 h dark), fed Purina Lab Chow and water ad libitum, unless otherwise indicated. The procedures of the study were in accordance with the guidelines of the Committee for the Use and Care of Laboratory Animals in Ben-Gurion University of the Negev, Israel (Authorization # IL-53-08-2020(E)). At the beginning of experiments, rats were randomly assigned to the different treatment groups. Changes were performed only to adjust for significant differences in average body weight of the groups.

### Behavioral tests

**Figure 2:**
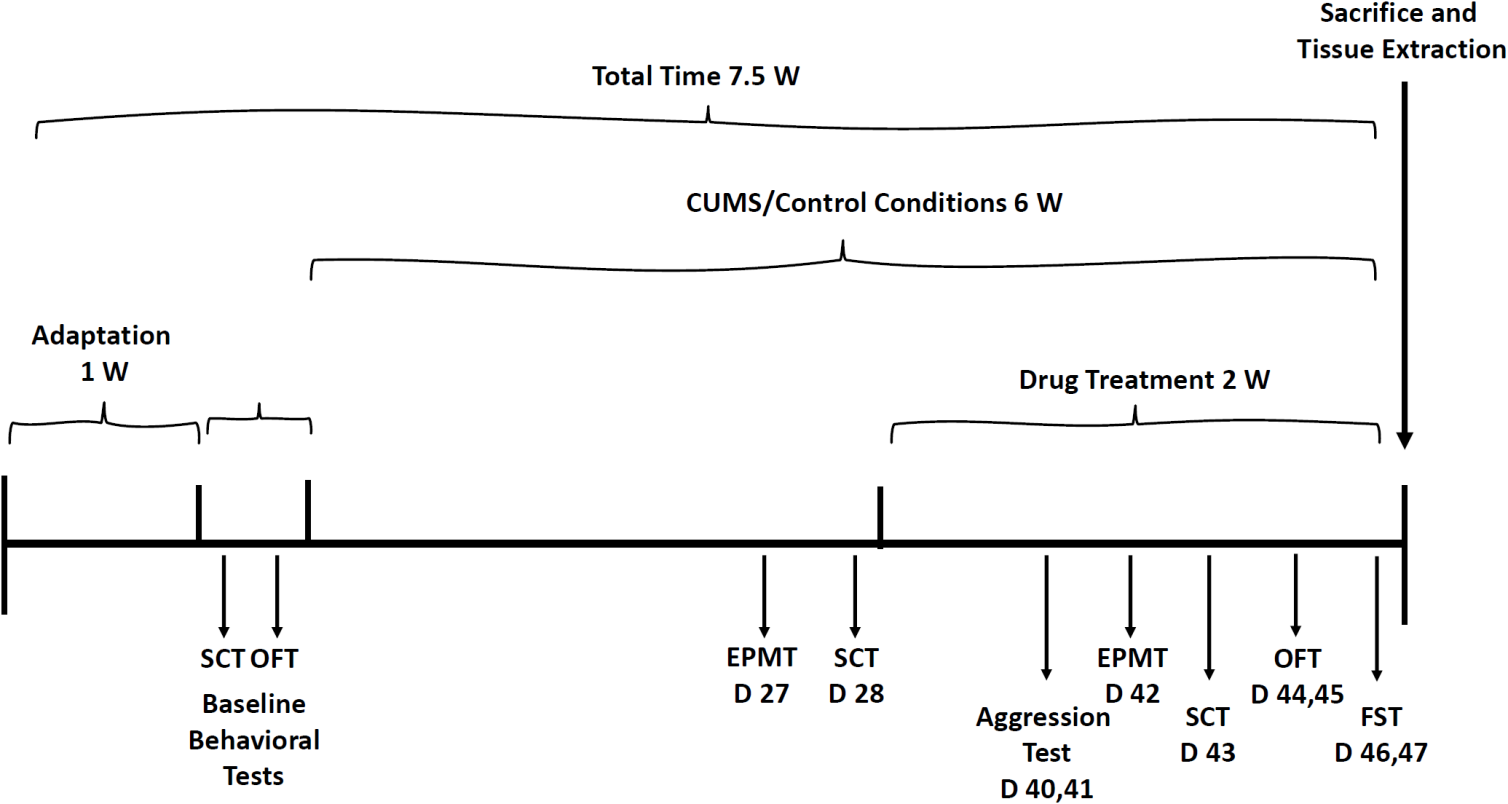
Timeline for behavioral experiments. Before initiation of the CUMS protocol, baseline SCT and OFT were performed. Then, rats were subjected to CUMS or control conditions for six weeks during the last two weeks of which, they were treated with MTK (or vehicle). The various behavioral tests were performed on different days of the experiment as illustrated in the figure. CUMS, chronic unpredictable mild stress; D, day; EPMT, elevated plus-maze test; FST, forced swim test; OFT, open field test; SCT, sucrose consumption test; W, week.

Albeit animal models in translational psychiatry research are limited, this study performed experiments by simulating the most widely recognized and phenotypically validated behavioral tests (81–87). Before initiation of behavioral studies, rats were accustomed to housing conditions for one week and then subjected to the different behavioral experiments. Depression-like phenotypes were induced by exposing male and female rats to a chronic unpredictable mild stress (CUMS) protocol (81–87) for four weeks. Thereafter, the following tests were conducted, at different time-points, to examine the effects of MTK on animal behavior: open field test, sucrose consumption test, elevated plus-maze test, aggression test and forced swim test. **Figure 2** presents the timeline of the behavioral experiments.

#### Chronic unpredictable mild stress paradigm

Prior studies have shown that external stressors may induce depressive-like behavior. Rats were subjected to a CUMS protocol to induce core symptoms of depressive-like behaviors, as described previously (81–87), with some modifications. The CUMS protocol was initiated following a one-week adaptation period and a three-day evaluation of baseline behavioral tests (total of one and a half weeks). The protocol extended for six weeks, the last two of which were concurrent with MTK (or vehicle) treatment. The CUMS protocol consisted of chronic exposure to different mild stressors every day for certain periods of time, including: group housing (six rats instead of three per cage; 8 h), placement in a tilted cage (30°, 3 h), food deprivation (11 h), water deprivation (11 h), placement in a soiled cage (5 h), and exposure to perfume odor (8 h) (81–87). At each time-point of the CUMS protocol, animals were simultaneously subjected to a maximum of two stressors, and with a maximum of three stressors per day (see **Table S1** for precise sequence and layout schedule of the stressors as conducted).

#### Open field test

The OFT was performed as described previously (85, 87–89). Rats were placed for 20 minutes in an open field arena. The arena is made of a black box (60 cm [W] × 80 cm [L] × 60 cm [H]) which was divided into a 25% central zone and a 75% peripheral zone. Rats were placed in the corner of the arena. Sessions were videotaped by a camera placed approximately one meter above the center of the arena and subsequently assessed using a video-tracking system (Etho-Vision XT 14; Noldus Information Technology, Netherland). A 5% ethanol in water solution was used to clean the arena prior to the introduction of each animal. The initial 10 minutes of each session were regarded as adaption time; thus, only the last 10 minutes of the sessions were analyzed. The parameters that were determined are: total distance traveled, mean velocity (of movement) and the percentage of time spent in the peripheral versus central zone of the arena. Calculation was as follows: (seconds spent in each zone/600) x 100. The OFT was conducted under similar conditions at two time points: before starting the CUMS protocol (baseline), and after two weeks of MTK treatment (days 44 and 45 of the protocol, see **Figure 2**).

#### Sucrose consumption test

This test is used to assess anhedonia – a behavioral feature of depression. The test was conducted as described previously (85, 87), with slight modifications. Sucrose consumption during a 10-hour session was calculated as described previously (85, 87). The SCT was conducted under similar conditions at three time points as described in **Figure 2**.

#### Elevated plus-maze test

This test intends to measure anxiety-like behavior (associated with depression) and risk-taking behavior (associated with mania). The test was performed as described previously (85, 87). The EPMT was conducted under similar conditions at two time points as indicated in **Figure 2**.

#### Aggression test

In this test, a naïve young-adult, stranger rat is brought to the cage of an adult investigational MTK/vehicle-treated rat. The rats were videotaped for 20 minutes and subsequently evaluated by three experienced observers who were blind to the treatment group of each animal. The number of physical attacks committed by the investigational rat against the naive rat was scored.

#### Forced swim test

The FST is a commonly used model for the assessment of depressive-like behavior in animals (81, 86, 90). The test examines immobility/floating time and struggling (climbing/diving) time. Immobility time represents despair/hopelessness and passive-like behavior. Struggling time represents active behavior. The test was conducted exactly as described previously (86).

### Treatment with MTK

After exposure to four weeks of the CUMS (or control) protocol, rats were treated intraperitoneally (ip), once daily, for two weeks with vehicle (dimethyl sulfoxide [DMSO] 0.1-0.2 ml/rat), or MTK (montelukast sodium; Sigma-Aldrich, St. Lewis, MO, catalog # PHR1603) 20 mg/kg, similar to several pharmacological studies that tested the effects of MTK *in-vivo* in rats (77, 91–94). MTK was dissolved in 100% DMSO and then administered at injection volumes that ranged between 0.1-0.2 ml according to rats’ body weight. Rats’ body temperatures and body weights were measured every third day before the administration of MTK/vehicle to verify that the treatment itself did not affect these vital measures.

### Brain sample collection

At the end of the treatment protocol, rats were briefly anesthetized (with 4% isoflurane in 100% oxygen) and immediately euthanized by decapitation. Then, brain regions (FC, HT, and HC) were excised, similar to previous studies (85–87).

### ELISA for measurement of IL-6, TNF-α and PGE2

Levels of IL-6, TNF-α and PGE2 in brain samples were detected using specific ELISA kits according to the manufacturer’s (R&D Systems, Minneapolis, MN, USA) instructions, exactly as described previously (85–87). The detection range of the assays was 125-8000 pg/ml for IL-6, 62.5-4000 pg/ml for TNF-α and 39-2500 pg/ml for PGE2. Samples in which the level of the examined cytokine was below the lowest detection limit of the assay were classified as “undetectable” and calculated as zero.

### Statistical analyses and presentation of data

Firstly, Shapiro-Wilk and Kolmogorov-Smirnov tests were used to verify data normality. Accordingly, statistical significance was determined by one-way ANOVA (with Benjamini-Hochberg false discovery rate) followed by Student’s t-test for normally distributed variables, and the Kruskal-Wallis test (with Benjamini, Krieger and Yekutieli false discovery rate) for abnormally distributed variables. Values of *P* < 0.05 were considered statistically significant. Results are shown as mean ± SEM (standard error of mean). We performed two independent experiments in male rats (n = 12 per group) and three independent experiments in female rats (n = 12 or 18 per group). In one of the “female experiments” we included 18 rats per group, because in the other two experiments we realized that the intra- and inter-group variability was high in some of the behavioral tests. Thus, we enlarged the sample size to increase reliability of the results obtained in the previous experiments. A typical experiment included the following four groups: 1) Control (not exposed to CUMS) + DMSO; 2) Control + 20 mg/kg MTK (dissolved in DMSO); 3) CUMS + DMSO; and, 4) CUMS + 20 mg/kg MTK.

## Results

### Behavioral tests

Results of the different behavioral tests examining the efficacy of MTK in male and female rats succeeding a six-week CUMS protocol are presented below.

#### MTK treatment reduces manic-like behaviour in male rats in the open field test

**Figure 3:**
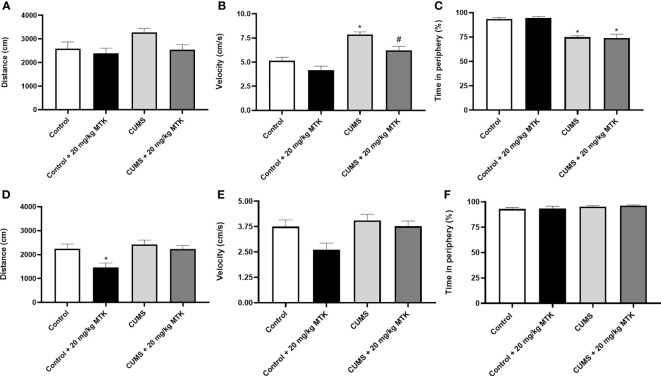
Effects of MTK on rats' behavior in the open field test. Male **(A-C)** and female **(D-F)** rats were subjected to CUMS or control conditions for six weeks. During the last two weeks of the protocol, animals were treated (ip) with MTK 20 mg/kg or vehicle. On days 44 and 45, rats were placed for 20 minutes in an open field arena; only the last 10 minutes of the session were assessed. The parameters that were analyzed are: total distance traveled **(A, D)**, mean velocity **(B, E)**, and the percentage of time spent in the peripheral zone **(C, F)**. Each column is the mean ± SEM of 12 to 18 rats per group. Using one-way ANOVA (with Benjamini-Hochberg false discovery rate) followed by Student’s t-test for normally distributed variables, and Kruskal-Wallis test (with Benjamini, Krieger and Yekutieli false discovery rate) for abnormally distributed variables, for specific group comparisons: *p < 0.05 vs. Control + DMSO; #p < 0.05 vs. CUMS + DMSO.

A baseline OFT was conducted before the initiation of the CUMS protocol revealing non-significant differences between the groups in terms of total distance traveled and mean velocity (data not shown). **Figure 3** presents the results of the OFT at the end of the experiment, after two weeks of MTK treatment. As seen, generally, all treatment conditions (CUMS vs. control, and MTK vs. vehicle treatment) did not significantly alter the total distance traveled by neither male nor female rats (**Figures 3A, D**). An exception is that MTK significantly decreased the distance traveled in female rats (**Figure 3D**). Moreover, male rats that were exposed to the CUMS protocol upturned a significantly increased velocity of movement, which was significantly reduced by MTK treatment, suggestive of an anti-manic/hyperactive-like effect (**Figure 3B**). In female rats, neither the exposure to CUMS nor the treatment with MTK led to significant differences in velocity (**Figure 3E**). Furthermore, there was a significant decrease in the time spent in the peripheral zone in males that were subjected to CUMS (**Figure 3C**), suggestive of a risk-taking/manic-like behavior. In females, the exposure to CUMS did not lead to a significant change in this measure (**Figure 3F**). Treatment with MTK did not alter this measure in neither male nor female rats (**Figures 3C, F**, respectively).

#### MTK treatment induces an antidepressant-like effect in the sucrose consumption test

**Figure 4:**
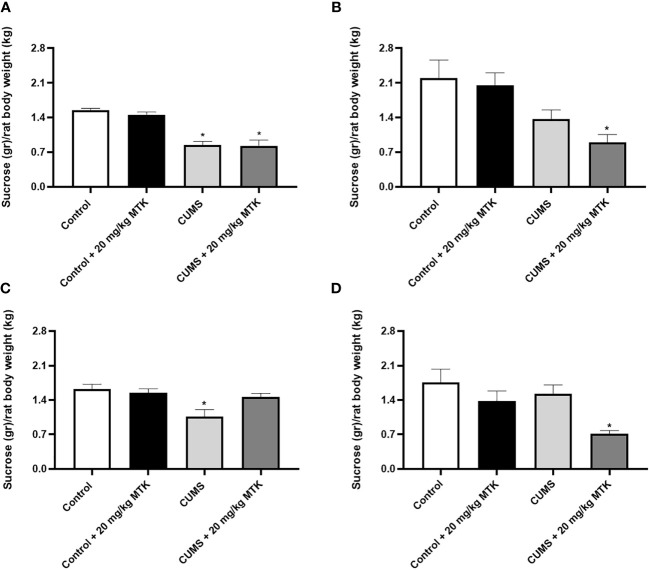
Effects of MTK on sucrose consumption. Male **(A, C)** and female **(B, D)** rats were exposed to CUMS or control conditions for four weeks **(A, B)** and then, while remaining under these conditions, were treated for two weeks with MTK 20 mg/kg or vehicle **(C, D)**. Each column is the mean ± SEM of 12 to 18 rats per group. Using one-way ANOVA (with Benjamini–Hochberg False Discovery Rate), followed by Student’s t-test for specific group comparisons: *p < 0.05 vs. Control + DMSO.

As seen in **Figure 4**, after four weeks of CUMS (before commencement of MTK treatment), male rats had a significantly decreased sucrose consumption as compared to control animals (**Figure 4A**), suggestive of depressive-like behavior. Treatment with MTK reversed the decrease in sucrose consumption in stressed males (**Figure 4C**). In females, after four weeks of CUMS, there was a non-significant decrease in sucrose consumption in one of the tested groups and a significant decrease in other (**Figure 4B**). These changes were not significantly affected by MTK treatment *(* **Figure 4D** *)*.

#### MTK treatment does not alter anxiety-like behavior in the elevated plus-maze test

**Figure 5:**
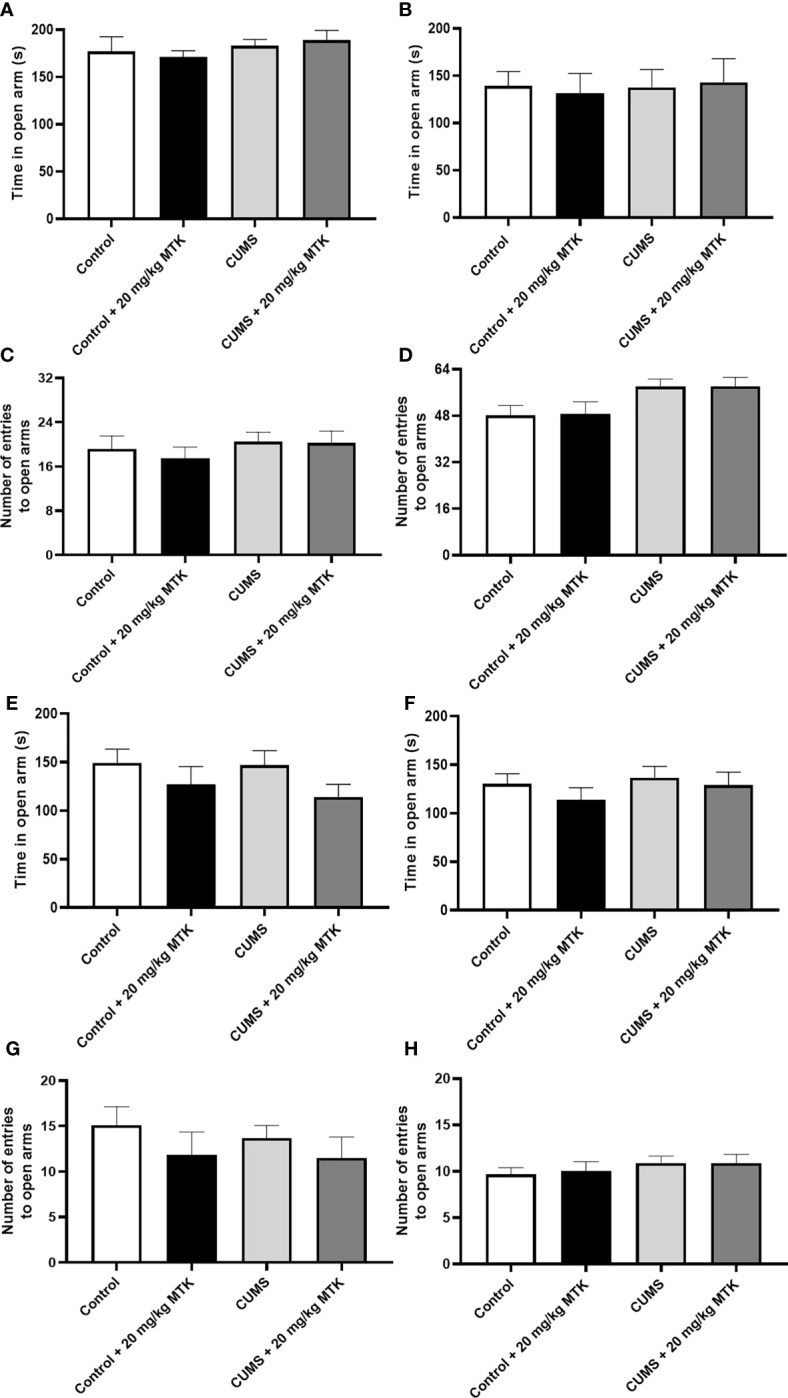
Effects of MTK on rats' behavior in the elevated plus-maze test. Male **(A, C, E, G)** and female **(B, D, F, H)** rats were exposed to CUMS or control conditions for four weeks **(A-D)** and then, while remaining under these conditions, were treated for two weeks with MTK 20 mg/kg or vehicle **(E-H)**. Time spent in open arms **(A, B, E, F)** and number of entries into the open arms **(C, D, G, H)** were evaluated. Each column is the mean ± SEM of 12 to 18 rats per group. Using one-way ANOVA (with Benjamini–Hochberg False Discovery Rate), followed by Student’s t-test for specific group comparisons, no significant differences between the groups were observed.

Exposure to CUMS for four weeks did not significantly influence the time spent in the open arms in neither male nor female rats (**Figures 5A, B**, respectively), nor did it significantly alter the number of entries into the open arms in male or female rats (**Figures 5C, D**, respectively). Two weeks of MTK treatment also did not affect the time spent in the open arms in male and female rats (**Figures 5E, F**, respectively), nor the number of entries into the open arms in male and female rats (**Figures 5G, H**, respectively).

#### MTK treatment invokes aggressive-like behavior in males but mitigates aggression in females

**Figure 6:**
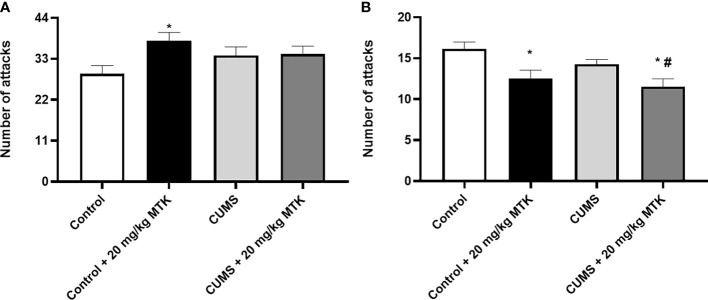
Effects of MTK on aggressive-like behavior. Male **(A)** and female **(B)** rats were exposed to CUMS or control conditions for four weeks and then, while remaining under these conditions, were treated with MTK 20 mg/kg or vehicle. Each column is the mean ± SEM of 12 to 18 rats per group. Using one-way ANOVA (with Benjamini–Hochberg False Discovery Rate), followed by Student’s t-test for specific group comparisons: *p < 0.05 vs. Control + DMSO; #p < 0.05 vs. CUMS + DMSO.

As seen in **Figure 6**, exposure to the CUMS protocol did not cause significant changes in the number of attacks committed by the investigational rat against the naïve rat both in males and females. Treatment with MTK increased the number of attacks in control male rats (**Figure 6A**), suggestive of a possible aggression-inducing effect of the drug. On the other hand, treatment with MTK significantly decreased the number of attacks in control as well as CUMS-subjected female rats (**Figure 6B**), indicative of an anti-aggressive-like effect of the drug.

#### MTK treatment does not induce depressive-like behavior in the forced swim test

**Figure 7:**
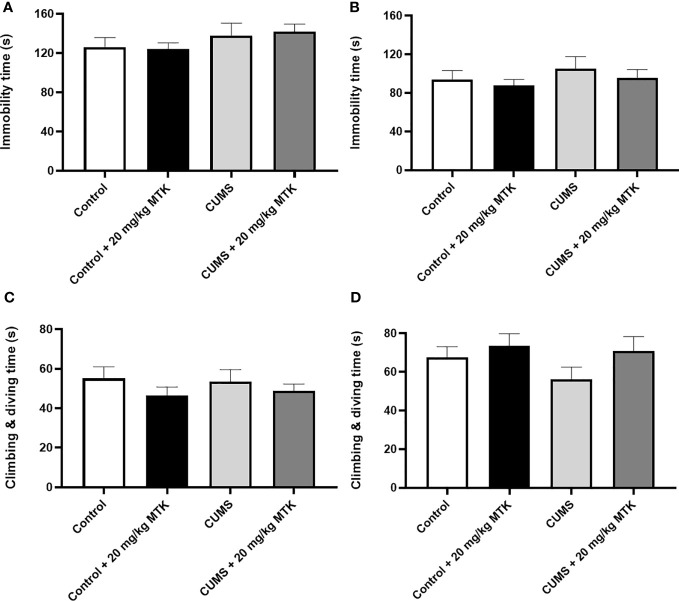
Effects of MTK on immobility and struggling time. Male **(A, C)** and female **(B, D)** rats were exposed to CUMS or control conditions for four weeks and then, while remaining under these conditions, were treated for two weeks with MTK 20 mg/kg or vehicle. Immobility time **(A, B)** and struggling (climbing/diving) time **(C, D)** were evaluated at the end of chronic MTK treatment. Each column is the mean ± SEM of 12 to 18 rats per group. Using one-way ANOVA (with Benjamini–Hochberg False Discovery Rate), followed by Student’s t-test for specific group comparisons, no significant differences between the groups were observed.

Surprisingly, exposure to CUMS for six weeks did not significantly influence the immobility time or struggling time in neither male nor female rats (**Figure 7**). Moreover, two weeks of MTK treatment did not alter the immobility time and struggling time in control and CUMS males and females (**Figures 7A–D**, respectively). These findings indirectly suggest that MTK does not seem to induce depressive-like behavior.

### Brain inflammation experiments

As mentioned in *Methods*, at the end of the MTK treatment protocol, rats were euthanized and brain regions (FC, HT and HC) were excised to determine the levels of the inflammatory mediators IL-6, TNF-α and PGE2.

#### MTK treatment significantly reduces brain IL-6 levels in female but not male rats

**Figure 8:**
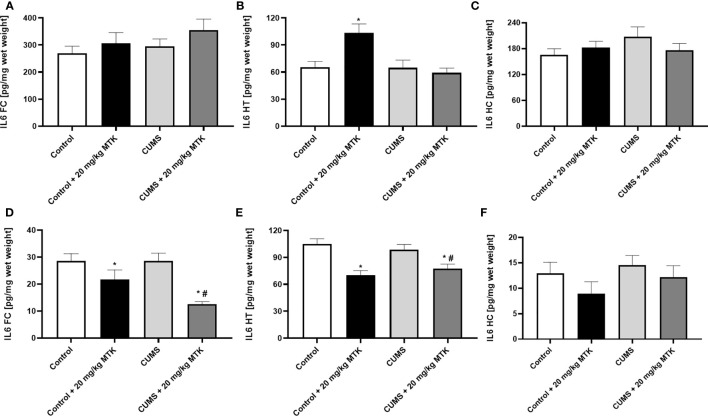
Effects of MTK on brain IL-6 levels. Male **(A-C)** and female **(D-F)** rats were exposed to CUMS or control conditions for four weeks and then, while remaining under these conditions, were treated for two weeks with MTK 20 mg/kg or vehicle. IL-6 levels in FC **(A, D)**, HT **(B, E)** and HC **(C, F)** were determined by ELISA. Each column is the mean ± SEM of 12 to 18 rats per group. Using one-way ANOVA (with Benjamini–Hochberg False Discovery Rate), followed by Student’s t-test for specific group comparisons: *p < 0.05 vs. Control + DMSO; #p < 0.05 vs. CUMS + DMSO.

**Figure 8** shows that exposure to CUMS for six weeks did not cause significant changes in IL-6 levels in the FC, HT and HC of male and female rats. Treatment with MTK was not associated with prominent effects on IL-6 levels in male rats (**Figures 8A–C**). Of note, MTK significantly increased IL-6 levels in the HT of control male rats (**Figure 8B**) but did not cause a prominent effect in the FC and HC (**Figures 8A, C**, respectively). In female rats, MTK treatment was mostly associated with a decrease in IL-6 levels (**Figures 8D–F**). For instance, MTK significantly reduced IL-6 levels in control (HT) and CUMS-subjected rats (FC & HT).

#### MTK treatment profoundly decreases TNF-α levels in female but not male rats

**Figure 9:**
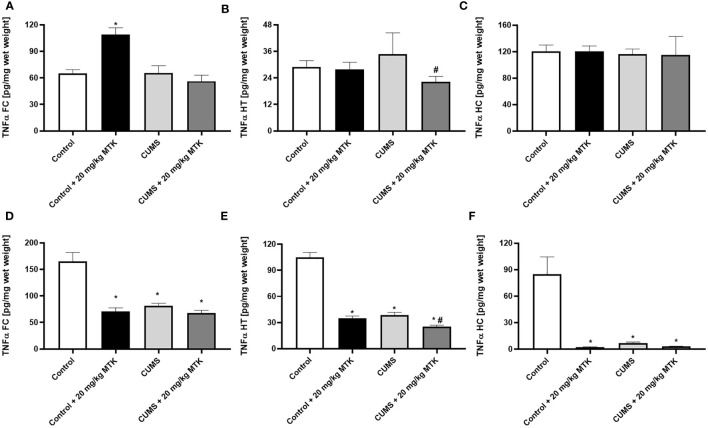
Effects of MTK on brain TNF-α levels. Male **(A-C)** and female **(D-F)** rats were exposed to CUMS or control conditions for four weeks and then, while remaining under these conditions, were treated for two weeks with MTK 20 mg/kg or vehicle. TNF-α levels in FC **(A, D)**, HT **(B, E)** and HC **(C, F)** were determined by ELISA. Each column is the mean ± SEM of 12 to 18 rats per group. Using one-way ANOVA (with Benjamini–Hochberg False Discovery Rate), followed by Student’s t-test for specific group comparisons: *p < 0.05 vs. Control + DMSO; #p < 0.05 vs. CUMS + DMSO.

**Figure 9** shows that there was a dramatic difference in brain TNF-α levels between male and female rats. In males, the exposure to CUMS did not significantly affect TNF-α levels in the FC, HT and HC (**Figures 9A–C**, respectively). There was no consistent trend in the effect of MTK on TNF-α levels in male rats: MTK significantly increased TNF-α levels in the FC in control animals but significantly decreased its levels in the HT of CUMS-subjected males (**Figures 9A, B**, respectively). Contrastingly, in females, the exposure to CUMS led to a significant decrease in TNF-α levels in the FC, HT and HC (**Figures 9D-F**, respectively). Moreover, MTK profoundly reduced TNF-α levels in control females in all brain regions but, mostly, did not further decrease its levels in MTK-treated females (**Figures 9D–F**).

#### MTK treatment differently affects PGE2 levels in the various brain regions

**Figure 10:**
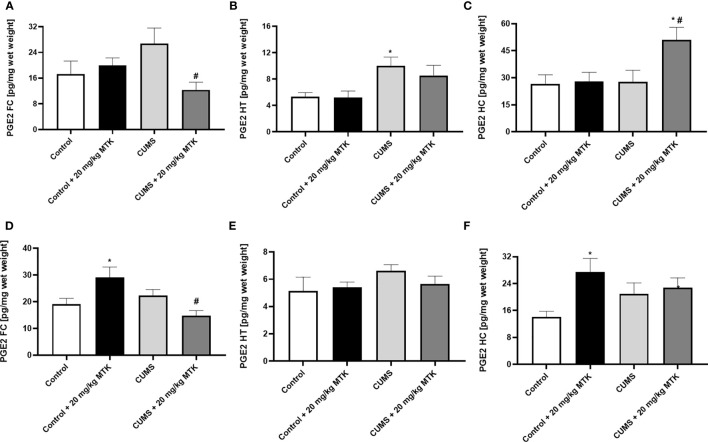
Effects of MTK on brain PGE2 level. Male **(A-C)** and female **(D-F)** rats were exposed to CUMS or control conditions for four weeks and then, while remaining under these conditions, were treated for two weeks with MTK 20 mg/kg or vehicle. PGE2 levels in FC **(A, D)**, HT **(B, E)** and HC **(C, F)** were determined by ELISA. Each column is the mean ± SEM of 12 to 18 rats per group. Using one-way ANOVA (with Benjamini–Hochberg False Discovery Rate), followed by Student’s t-test for specific group comparisons: *p < 0.05 vs. Control + DMSO; #p < 0.05 vs. CUMS + DMSO.

**Figure 10** shows that the exposure to CUMS for six weeks did not lead to significant changes in brain PGE2 levels in male (except in HT) and female rats. There was no consistent trend in the effect of MTK on PGE2 levels in male rats: MTK significantly decreased PGE2 levels in the FC and HT of CUMS-subjected males (**Figures 10A, B**, respectively), but significantly increased its levels in the HC (**Figure 10C**). Similarly, in females the effects of the drug on PGE2 levels were different in the various brain regions (**Figures 10D-F**). MTK significantly elevated PGE2 levels in the FC (*D*) and HC (*F*) of control females but reduced its levels in FC of CUMS-subjected females (*D*). On the other hand, MTK did not alter PGE2 levels in the HT of female rats (**Figure 10E**).

## Discussion

The main objective of the present study was to directly examine the effects of MTK on the behavioral phenotype of male and female rats. This is because several studies reported that MTK may induce ANPEs in treated patients (66–68, 79, 95, 96). Under the experimental conditions of the present study, we found that MTK does *not* cause prominent negative behavioral effects in rats. **Table 1** summarizes the effects of MTK (20 mg/kg) on the behavior of male and female rats as evaluated in the utilized behavioral models. It is seen that, generally, MTK did not adversely influence animal behavior in either control (non-stressed) or CUMS-subjected rats (**Table 1**). An exception was the aggression-inducing effect of MTK in male rats (**Figure 6A** and **Table 1**). However, this negative effect of MTK is “counter-balanced” by its positive behavioral effects in males and females in other conditions. Together, within the scope of the particular experimental conditions applied, a generally safe behavioral profile of MTK in male and female rats was observed. This is consistent with the findings of several recent established studies that examined the association between MTK treatment and the occurrence of ANPEs in human subjects (67, 97, 98). Nonetheless, for the sake of scientific veracity, it has to be noted that other recent established studies revealed contradicting findings, *i.e.*, that MTK increases the occurrence of ANPEs (73, 99, 100).

**Table 1:** Effect of MTK on behavior.

		Effect of MTK in control rats		Effect of CUMS (alone)		Effect of MTK in CUMS-subjected rats	
		Male	Female	Male	Female	Male	Female
**OFT**	**Velocity**	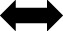	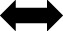	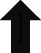	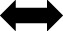	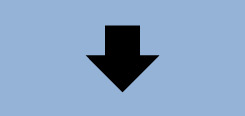	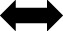
	**Distance traveled**	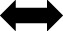	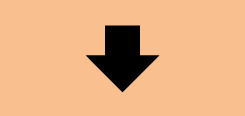	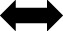	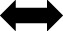	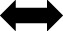	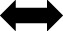
	**Time in periphery**	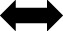	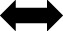	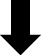	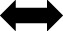	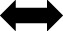	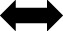
**SCT**		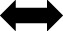	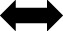	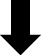	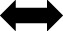	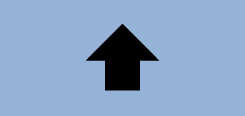	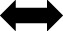
**EPMT**	**Time in open arms**	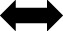	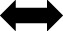	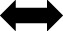	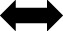	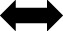	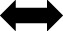
	**Number of entries to the open arms**	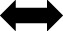	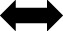	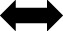	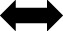	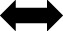	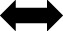
**Aggression**		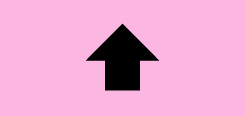	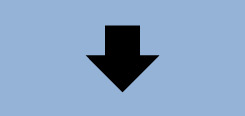	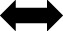	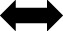	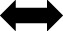	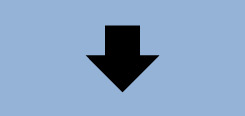
**FST (immobility)**		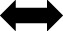	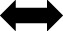	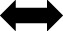	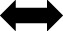	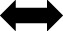	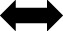

The table illustrates the effects of the treatment protocol on different behavioral phenotypes. The signs indicate the following trends: 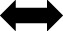
- a non-significant effect;
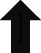
- an increase, 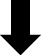
- a decrease; pink- a possible harmful effect of MTK; blue - a possible beneficial effect of MTK; orange - a disputable effect of MTK. CUMS, chronic unpredictable mild stress; EPMT, elevated plus-maze test; FST, forced swim test; MTK, montelukast; OFT, open field test; SCT, sucrose consumption test.

LTs are inflammatory mediators that strongly affect the function of mammalian tissues including the brain (28). Considering the pro-inflammatory effects of LTs and the large body of data which suggests that inflammation contributes to the pathophysiology of mental disorders (4, 6, 7, 15, 22), we hypothesized that treatment with MTK – a potent cys-LTs receptor antagonist – might exert beneficial behavioral effects in psychiatric disorders (31, 32, 75). Indeed, MTK incited some beneficial pre-clinical behavioral outcomes in rats (**Table 1**): 1) MTK treatment increased sucrose consumption in stressed male rats, suggestive of an antidepressant-like effect. 2) MTK reduced the hyperactive/manic-like phenotype in CUMS-subjected males (as modeled in the OFT), including a reduction in mean velocity and reversal of the decreased time spent in the peripheral zone of the arena. In this regard, when placed into an open field, rats and mice tend to remain in the peripheral zone of the arena or against the walls (88, 89). To the best of our knowledge, this is the first study showing a significant anti-hyperactive/anti-manic-like effect of MTK in animals. 3) MTK mitigated aggressive-like behavior in female rats. These findings support our hypothesis that MTK might capacitate beneficial behavioral effects, similar to other typical anti-inflammatory medications such NSAIDs and corticosteroids (15–21). Recently, Tel *et al.* (101) showed that MTK exhibited antidepressant-like effects (assessed by measuring immobility time in the FST) in male and female mice that were subjected to an ovalbumin-induced asthma model. MTK significantly decreased immobility time in the ovalbumin-challenged, but not in control mice (101). These findings resemble the results that we obtained in the SCT, where MTK treatment did not alter sucrose consumption in control animals but reversed the reduction of sucrose consumption in CUMS-subjected male rats (**Figure 4C** and **Table 1**). Another LTMA, the 5LOX inhibitor zileuton, was also found to decrease immobility time in a lipopolysaccharide induced depression-like model in mice (102), supporting the suggested possible anti-depressive effect of LTMAs. Furthermore, aggressiveness and self-harm were among the most commonly reported ANPEs of MTK (66–68, 95). In the present study, MTK increased aggressive-like behavior in male rats but decreased it in females (**Figure 6** and **Table 1**), suggestive of a sex-associated effect of the drug. These results are in line with those of a previous study which demonstrated that LTMAs were pharmacologically effective in female mice but not males (56), and may further reinforce conjectures of sex-related variance in pharmacotherapeutic responses among psychiatric patients (103). Of note, it is difficult to explain and categorize the MTK-induced reduction in distance traveled in control female rats (**Figure 3D** and **Table 1**) as a therapeutic or harmful effect. This is because, on one hand, in this animal model this outcome is usually interpreted as an anti-manic/hyperactive-like effect of a given treatment intervention. However, on the other hand, since their “basal/normal” behavior was altered (compared to vehicle-treated control females, which is the “pure” control group in this setting), it can be argued that it represents an undesirable effect of MTK.

Furthermore, we tested the behavioral effects of MTK in the EPMT which models anxiety-like behavior. Rodents are inherently inclined to seek dark hiding spaces during light hours (in the present study the test was conducted during light hours) in attempt to evade being seen by predators. Therefore, remaining in the closed arms of the maze is considered characteristic normative behavior, if the time spent in these arms is proportionally comparable to that of the control animals. However, when the time rats’ dwell in the open arms significantly exceeds that of the closed arms, it is generally interpreted as *non*-anxious behavior. Contrastingly, if the time situated in the open arms markedly surpasses that observed among control rats, it may denote risk-taking/manic-like behavior. The results show that exposure to CUMS did not cause behavioral changes in the EPMT in male and female rats (**Figure 5** and **Table 1**). Moreover, treatment with MTK, both in control and CUMS-subjected rats, did not alter animals’ behavior in the EPMT. This clearly indicates that, under these experimental conditions, MTK did not induce anxious-like behavior.

Interestingly, we found that there were differences in the influential intensity of CUMS on the behavioral phenotypes of male vs. female rats (**Table 1**). For example, in CUMS-subjected male rats there was a significant increase in mean velocity and a decrease in time spent the peripheral zone of the arena (both suggestive of manic-like behavior), while no such changes were observed in CUMS-subjected females. Additionally, the exposure to CUMS led to a significant decrease in sucrose consumption (indicative of depressive-like behavior) in males, while in females CUMS was associated with only a nearly significant decrease in sucrose consumption (**Figure 4**). This is consistent with the results of previous studies which showed that stress protocols induce a reduction in sucrose preference/consumption only in male rats (104–106). However, other studies reported opposite results, *i.e.*, that exposure to stress protocols induces a significant reduction in sucrose preference/consumption only in females (58, 107), or causes a decrease in males as well as females (108, 109). Furthermore, several previous studies reported that various stress protocols led to a significant increase in immobility time in the FST, suggestive of depressive-like behavior (81–84). Surprisingly, in the present study the utilized CUMS protocol did not cause a significant change in immobility time (**Figure 7** and **Table 1**). Our results are similar to those of previous studies (105, 110) which also demonstrated that some stress protocols do not induce significant changes in immobility time in male and female rats. It is known that the type, severity and duration of the stress protocol, and, the strain and sex of used animals all affect the measured behavioral outcomes (81–84, 106, 110–112).

The current study used subjects of the female sex heterogeneously spanning across different time points in the estrous cycle throughout experiments, with normal distribution applied similarly across all randomized female study cohorts. While we did not perform direct analytical or descriptive evaluation contingent upon the females’ hormonal cycles, we did ensure that sample sizes contained an ample number of subjects in order to neutralize potential deviations that may have related to such. Nevertheless, we acknowledge this as a limitation of our study, as research shows demonstrable correlations between inflammatory biomarkers, AA metabolites, the corpus luteum, and expressive ovulatory and implantation mechanisms (113–117). Pertinent to the behavioral experiments of the present study, Zhao *et al.* (118) investigated sex-specific depression and the LTA4 hydrolase haplotype gene (an aminopeptidase dictating the conversion of LTA4 to LTB4). Their findings show gene expression to confer with a significant protective effect in women in regard to depression, rationalizing further examination of cys-LTs and sex differences in mental illness. Importantly, MTK treatment has been reported to affect endometrial conditions (119) as well as other female hormonal aspects (57, 120, 121). For example, MTK was found to exert better control of asthmatic symptoms in women than men (57). Furthermore, Fujiwara *et al.* (121) found in a randomized, double-blind, placebo-controlled trial that MTK was effective in reducing dysmenorrheal pain in women. Numerous studies reported on the bidirectional association between LT function and the estrous cycle and reproductive system (116, 122–124). Therefore, it is feasible that the blatant sex-associated differences in our study are affiliated with the relating female hormonal processes involved, and stratified analyzation of this parameter may have yielded interesting outcomes.

As mentioned, a large body of data suggested that inflammation contributes to the pathophysiology of mental disorders (15, 22, 125–132). For example, several research papers reported increased IL-6 levels in patients with major depression (129, 130, 133), bipolar disorder (129, 131) and schizophrenia (129, 130). Moreover, multiple studies showed that IL-6 levels were prominently increased in the blood of subjects after suicidal attempts and in post-mortem brains of people after suicidal death (134–136). Similarly, many studies found that TNF-α levels are higher in patients with major depression (129, 133), bipolar disorder (129, 131, 137) and schizophrenia (129, 137) than in matched-controlled subjects. PGs (PGE2 in particular) have been recurrently observed as connected to the pathophysiology of psychiatric disorders (138, 139). These outcomes are highly relevant to the behavioral findings of the present work, because numerous studies have demonstrated that MTK decreases the levels of several pro-inflammatory mediators including IL-6, TNF-α and PGE2 under various experimental conditions (34, 37, 40, 42, 140, 141). Thus, we hypothesized that the behavioral effects of MTK may be influenced by and related to its effects on brain inflammation. In the present study we assessed brain inflammation by measuring inflammatory mediator levels in the FC, HT and HC (11–13). **Table 2** summarizes the effects of MTK treatment and the exposure to CUMS on levels of IL-6, TNF-α and PGE2 in these brain regions. As seen, in control males, MTK increased IL-6 levels in the HT, and TNF-α levels it the FC, suggestive of a *pro*-inflammatory effect of the drug. In contrast, in control females, MTK treatment was associated with a robust *anti*-inflammatory effect; it significantly decreased IL-6 and TNF-α levels almost in all brain regions. On the other hand, MTK increased PGE2 levels in the FC and HC. These results are similar to those of previous studies which revealed that under certain conditions MTK may increase the production of PGE2 (44, 142). We speculate that the oppositional impact of MTK on IL-6 and TNF-α levels in male vs. female rats may contribute, at least in part, to its distinctive effect on aggressive-like behavior (**Table 1**). MTK induced aggressive-like behavior in males, while it decreased this behavior in females. Numerous studies demonstrated that aggressive behavior in humans and rodents is affected by the function and structure of the FC, HT and HC (143–145). Importantly, MTK also attenuated aggressive-like behavior in CUMS-subjected females (**Table 1**), and here too, there was a prominent reduction in TNF-α levels in the three brain regions and in IL-6 levels in the FC and HT (**Table 2**). In CUMS-subjected male rats the effect of MTK on inflammatory mediator levels was inconsistent and difficult to interpret. As for the effect of CUMS (alone) on inflammatory mediator levels, we found that the stress protocol did not alter brain levels of either IL-6, TNF-α or PGE2 (except in the HT) in male rats. In contrast, in females, the exposure to CUMS was associated with a profound reduction in TNF-α levels in all brain regions but particularly in the HC (**Figure 9**). A prominent reduction in TNF-α levels is usually considered an anti-inflammatory effect of a given intervention. However, again, since in this case it is a deviation from the basal/control condition, this interpretation may be disputable.

**Table 2:** Effect of MTK on brain inflammatory mediators’ levels.

		Effect of MTK in control rats		Effect of CUMS (alone)		Effect of MTK in CUMS-subjected rats	
		Male	Female	Male	Female	Male	Female
**FC**	**IL-6**	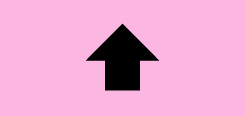	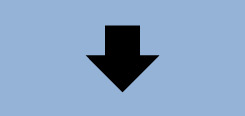	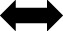	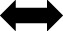	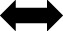	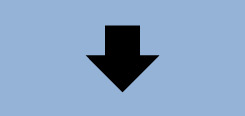
	**TNF-α**	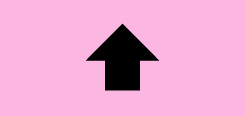	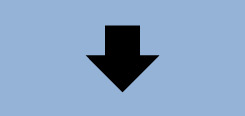	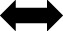	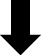	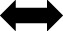	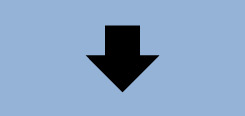
	**PGE2**	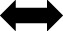	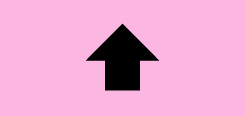	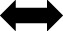	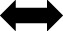	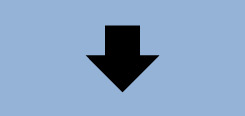	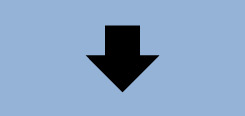
**HT**	**IL-6**	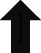	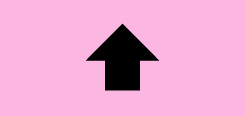	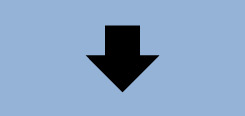	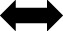	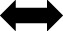	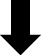
	**TNF-α**	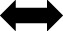	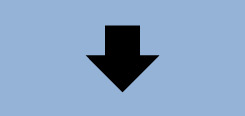	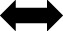	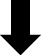	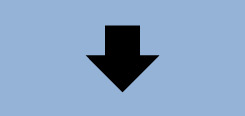	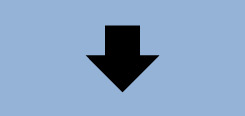
	**PGE2**	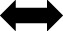	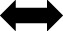	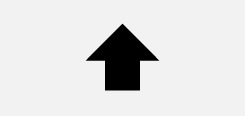	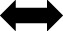	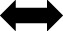	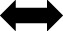
**HC**	**IL-6**	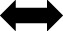	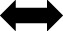	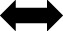	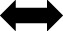	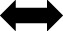	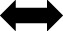
	**TNF-α**	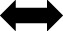	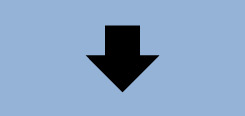	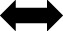	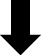	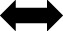	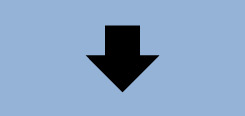
	**PGE2**	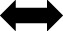	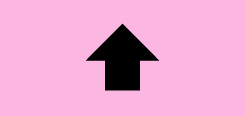	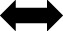	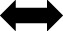	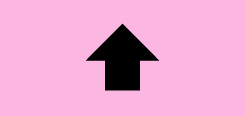	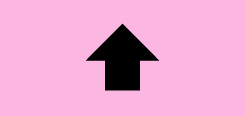

The table illustrates the effect of the treatment conditions on the various inflammatory mediators in the three examined brain regions: FC, HT and HC. The signs indicate the following trends: 
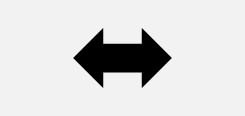
- a non-significant effect;
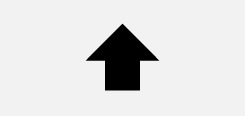
- an increase, 
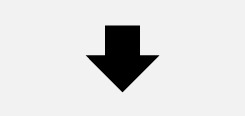
- a decrease; pink - a pro-inflammatory effect of MTK; blue - an anti-inflammatory effect of MTK. CUMS, chronic unpredictable mild stress; FC, frontal cortex; HC, hippocampus; HT, hypothalamus; IL-6, interleukin-6; MTK, montelukast; PGE2, prostaglandin E2; TNF-α, tumor necrosis factor-α.

To the best of our knowledge, this is the first study that directly and thoroughly tested the behavioral effects of MTK in rats. Overall, the results indicate that MTK treatment does not induce prominent adverse behavioral effects and may instead be associated with select beneficial behavioral outcomes. Moreover, the results support our hypothesis that treatment with MTK differentially affects levels of brain inflammatory mediators in male vs. female rats, which plausibly explains the dissimilar behavioral phenotypes of the sexes. Randomized, double-blind clinical trials in human subjects are necessary to directly determine the behavioral effectual capacity of MTK.

## Data availability statement

The primary data used to support the findings of this study are available from the corresponding author upon reasonable request.

## Ethics statement

The animal study was reviewed and approved by Committee for the Use and Care of Laboratory Animals in Ben-Gurion University of the Negev, Israel.

## Author contributions

Study concept and design – AA. Acquisition, analysis, or/and interpretation of the data – IR, BB-A, AN, ER, SU, JK, LB, AA. Statistical analysis – IR, SU, AA. Drafting of the manuscript – IR. Critical revision of the manuscript – All authors. Writing of the final version of the manuscript – AA. All authors approved the final version of the manuscript.

## Funding

This study was supported by a grant from the *Israel Science Foundation* (Grant # 198/12) to AA.

## Conflict of interest

The authors declare that the research was conducted in the absence of any commercial or financial relationships that could be construed as a potential conflict of interest.

## Publisher’s note

All claims expressed in this article are solely those of the authors and do not necessarily represent those of their affiliated organizations, or those of the publisher, the editors and the reviewers. Any product that may be evaluated in this article, or claim that may be made by its manufacturer, is not guaranteed or endorsed by the publisher.
