# Supplementary material for: Montelukast induces beneficial behavioral outcomes and reduces inflammation in male and female rats
Source: Front Immunol. 2022 Sep 6;13:981440. doi: 10.3389/fimmu.2022.981440 (PMC9487911; doi:10.3389/fimmu.2022.981440)
Supplement: Supplementary file 1 [file Table_1.docx]

|  | **Week 1** | **Week 2** | **Week 3** | **Week 4** | **Week 5** | **Week 6** |
| --- | --- | --- | --- | --- | --- | --- |
| **Stressor 1** | Cage tilt | Soiled cage | Perfume odor | Cage tilt | Soiled cage | Perfume odor |
| **Stressor 2** | Group housing | Cage tilt | Group housing | Soiled cag | Cage tilt | Group housing |
| **Stressor 3** | Water deprivation | Food deprivation | Cage tilt | Perfume odor | Water deprivation | Food deprivation |

Table S1. Chronic unpredictable mild stress (CUMS) protocol. The CUMS protocol consisted of chronic exposure to different mild stressors every day for certain periods of time. Each stressor is administered for 3-11 h, including: group housing (six rats instead of three per cage; 8 h), placement in a tilted cage (30°, 3 h), food deprivation (11 h), water deprivation (11 h), placement in a soiled cage (5 h), and exposure to perfume odor (8 h). Animals undergoing CUMS are transported daily to a clean room used for CUMS manipulations. Animals were simultaneously subjected to a maximum of two stressors, and to a maximum of three stressors per day. At the end of each daily stress period, animals were placed into clean cages and returned to the housing facility.
